# Power system low delay resource scheduling model based on edge computing node

**DOI:** 10.1038/s41598-023-41108-2

**Published:** 2023-09-05

**Authors:** Ying Zhao, Hua Ye

**Affiliations:** Yunnan Electric Power Grid Company, Kunming, 650011 Yunnan China

**Keywords:** Engineering, Mathematics and computing

## Abstract

As more and more intelligent devices are put into the field of power system, the number of connected nodes in the power network is increasing exponentially. Under the background of smart grid cooperation across power areas and voltage levels, how to effectively process the massive data generated by smart grid has become a difficult problem to ensure the stable operation of power system. In the complex calculation process of power system, the operation time of complex calculation can not be shortened to the greatest extent, and the execution efficiency can not be improved. Therefore, this paper proposes a two-phase heuristic algorithm based on edge computing. In solving the virtual machine sequence problem, for the main partition and the coordination partition, the critical path algorithm is used to sort the virtual machines to minimize the computing time. For other sub-partitions, the minimum cut algorithm is used to reduce the traffic interaction of each sub-partition. In the second stage of the virtual machine placement process, an improved best fit algorithm is used to avoid poor placement of virtual machines across physical machine configurations, resulting in increased computing time. Through the experiment on the test system, it is proved that the calculation efficiency is improved when the coordinated partition calculation belongs to the target partition. Because the edge computing is closer to the data source, it can save more data transmission time than cloud computing. This paper provides an effective algorithm for power system distributed computing in virtual machine configuration in edge computing, which can effectively reduce the computing time of power system and improve the efficiency of system resource utilization.

## Introduction

With the development of Internet of Things (IoT) to Internet of Everything (IoE), more and more intelligent devices are put into the field of power system, resulting in an exponential increase in the number of connected nodes in the power network. At the same time, the contemporary smart grid requires to realize the cooperation across power areas and voltage levels, and monitor the real-time status of each node of the power grid. However, there are a large number of nodes in the power system. How to process the massive data generated by them at a high speed and effectively has become a new challenge to ensure the stable operation of the power system^1^.

Massive data through cloud computing and a centralized computing method will bring a lot of transmission consumption and time delay problems^2^. Different from the traditional centralized computing mode, the edge computing application is deployed in the base station close to the terminal. In this computing mode, the server response and reliability are higher than those of the centralized big data processing mode, which can effectively reduce the bandwidth pressure and overload caused by massive data transmission^3^. Moreover, the intelligent devices in the edge network have abundant computing and storage resources, which can greatly reduce service delay and improve the quality of network service. However, the application of edge computing in the power system only stays in solving the preliminary calculation, and fails to organically combine the virtual machine configuration with the characteristics of the power system^4^. Most of the existing studies do not consider the structural characteristics of the power system, and ignore the impact of different information interaction requirements among system nodes on Virtual Machine Placement (VMP), which makes it impossible to give full play to the sufficient parallel computing and high-speed interaction capabilities of edge base stations in the complex computing process of the power system^5^.

For the research on virtual machine configuration, literature^6^ proposes a new load balancing algorithm from the perspective of load balancing, which configures virtual machines reasonably based on the number and size of incoming tasks to maximize the utilization of computing resources. On this basis, literature^7^ further clearly points out that the goal of virtual machine configuration is to minimize the waiting time and completion time of tasks. Inappropriate virtual machine configuration strategy will cause load imbalance between virtual machines, resulting in an increase in the total time to complete tasks. Literature^8^ and literature^9^ propose resource optimization allocation algorithms for the problem of manufacturing resource optimization allocation without demand preference in cloud manufacturing environment, considering manufacturing service demand and cloud platform operators. Reference^10^ proposes a GraspCC-fed algorithm to configure the optimal number of resources for each workflow to improve workflow performance and save costs for cloud data center virtual machine clusters. The methods proposed in these references have certain limitations in virtual machine configuration, mainly including the following aspects: Limitations of load balancing algorithms: The load balancing algorithm proposed in reference^6^ can allocate virtual machine resources reasonably based on the number and size of tasks, thereby maximizing the utilization of computing resources. However, this algorithm may not meet the performance and efficiency requirements of virtual machine configuration, as it only focuses on load balancing and ignores other key indicators such as task waiting time and completion time.Limitations on virtual machine configuration goals: Reference^7^ clearly states that the goal of virtual machine configuration is to minimize the waiting time and completion time of tasks. However, in practical applications, virtual machine configuration often requires consideration of more factors, such as resource utilization, energy conservation, and cost. Failure to fully consider these factors may lead to limitations in the configuration strategy, thereby affecting overall computing performance and resource utilization efficiency. Method limited to specific environments: Reference^10^ proposes a resource optimization configuration algorithm for manufacturing resources optimization in cloud manufacturing environments, combining manufacturing service requirements and cloud platform operators. However, this method is suitable for specific manufacturing environments and cannot be directly applied to other fields, such as distributed computing in power systems. Therefore, we need to design and develop suitable virtual machine configuration algorithms tailored to the specific needs and characteristics of the power system. Similarly, efficient computing power is also the goal of power system computing, so it is urgent to propose a virtual machine placement algorithm for power system distributed computing.

In this paper, considering the urgency of power system tasks, the tasks are divided into two categories: those that must be executed locally and those that can be migrated. On this basis, a time model is built to minimize the time consumption of task computation to clarify the objectives and constraints, and facilitate the subsequent evaluation of the effectiveness of the proposed algorithm. Then the attribution of the calculation amount of the coordinated partition of the interconnected power grid is divided by using the method of site selection in graph theory. The best regional power grid accommodating the coordinated partition is selected, so that the execution time of the task is improved. Finally, two kinds of most commonly used traditional virtual machine configuration algorithms, descending best fit algorithm and hierarchical clustering algorithm, are analyzed, and combined with the characteristics of power system. A two-phase heuristic algorithm for parallel distributed computing in power system is proposed. Without considering the task migration, the decomposition and coordination algorithm is used to partition the regional power grid. The effect of the proposed algorithm is compared with the traditional descending best fit algorithm and hierarchical clustering algorithm in terms of computing time and energy consumption.

With the Exponential growth of the number of connected nodes in the smart grid, how to effectively process the massive data from smart devices has become an important issue. This paper focuses on solving this challenge and proposes an algorithm based on edge computing, which can improve the efficiency of power grid data calculation and resource utilization.

This paper proposes a two-stage heuristic algorithm combining the Critical path method algorithm and the Minimum cut algorithm. In the first stage, the Critical path method algorithm is used to sort the virtual machines to reduce the computing time. In the second stage, an improved best match algorithm is used for virtual machine placement to avoid improper physical machine configuration and further optimize calculation time.

The innovation of this paper is to introduce edge computing technology into power system distributed computing. Edge computing is more close to the data source, so it can reduce the data transmission time and improve the computing efficiency. The experiment on the test system proves the effectiveness and advantages of edge computing in power system data processing.

## Related work

### Undirected weighting theory

In the addressing problem, an undirected weighted non-complete graph $G = (V,E)$, where $V(G) = \left\{ {v_{1} ,v_{2} , \ldots ,v_{n} } \right\}$ is the vertex set of *G*. $v_{i} \in V(i = 1,2, \ldots ,n)$ is the vertex of *G.* $E(G) = \left\{ {e_{1} ,e_{2} , \ldots ,e_{n} } \right\}$ is the edge set of *G* and $e_{ij} \in E(i,j = 1,2, \ldots ,n)$ is the edge from vertex $v_{i}$ to vertex $v_{j}$^11^. The relevant definitions are given below.

1. *Distance* The shortest distance between vertex *i* and vertex *j* in the graph *G* is the distance from vertex *i* to vertex* j* in the graph, denoted by $d_{ij}$ or $d(i,j)$; the distance from the point* f* on the arc $(r,s)$ to the vertex* r* of the arc is $f\;d(r,s)$, where $0 \le f \le 1$; the shortest distance from the point *f* on the arc $(r,s)$ to vertex *i* can be called the vertex-to-vertex distance, denoted by $d(f(r,s),i)$^12^.
2. *Median point* Let $SVV(i)$ denote the sum of the distances from vertex *i* to all other points in graph *G*. Searching for the vertex that minimizes $SVV(i)$ in all $i(i = 1,2, \ldots ,n)$ is called the median point of graph *G*^13^.

### Improved best fit algorithm

In order to meet the rationality of physical resource allocation, it is necessary to set corresponding rules for virtual machines:

Rule 1 When a certain sequence is configured, the remaining space of the physical machine is filled by searching its own sequence virtual machine in priority order.

Rule 2 When the undirected graph *G* cannot be cut into two connected subgraphs, the virtual machines of other sequences are searched in reverse order, and the virtual machines with the least traffic correlation of other partitions are used as far as possible^14^.

Rule 3 Multiple virtual machines in a connected subgraph are a continuous sequence of virtual machines in $VM_{list}$.

Rule 4 After obtaining $PM_{list}$ and $VM_{list}$, the remaining space resource $PM_{m \cdot spare}$ of the physical machine first searches the virtual machine sequence $VM_{m \cdot spare}$ of the connected subgraph as a whole. If $VM_{m \cdot spare} < PM_{m \cdot spare}$ is met, the sequence is placed in the physical machine. If not, other virtual machine sequences are placed from small to large according to the flow^15^.

After two stages of algorithm, the virtual machine configuration is completed. The first stage of the algorithm is to sort the virtual machines to obtain the virtual machine release sequence, which is essentially to prepare for the second stage of algorithm. Therefore, the algorithm is named Improved Best Fit (IBF)^16^.

## Task execution time model and two-stage heuristic algorithm

### Task execution time model

**Figure 1:**
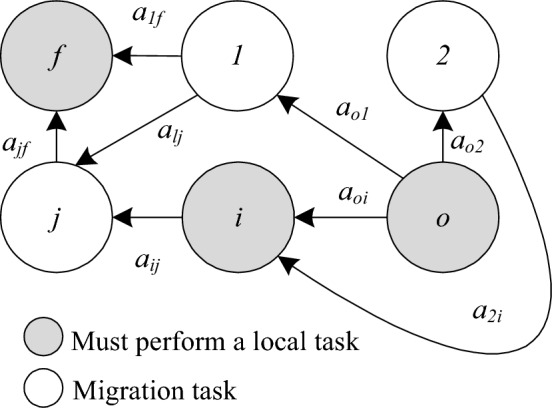
Task topology model.

Figure 1 is a task topology model, which uses a directed graph $G = (V,A)$ to represent the relationship between several independent tasks.

Each vertex $v \in V$ in Fig. 1 represents each task. The directed arc $a_{uv} \in A$ in the figure represents the data transferred between tasks (unit: bits). For example, $a_{ij}$ represents that the data of $a_{ij}$ will be transmitted to task *j* after task *i* is executed. Task* j* will not start execution until it receives the data transmitted after task *i* is executed^17^.

The tasks in Fig. 1 can be divided into two types: the first type is the task that must be executed locally, such as tripping caused by overload, which needs to be handled in time, and is represented as a solid node; the other type is the task that can be migrated, which is represented as a hollow node.

In this paper, a binary quantity $J_{uv} \in \left\{ {0,1} \right\}$ is defined to represent the line order of execution between tasks:

1 $$J_{uv} = \left\{ {\begin{array}{*{20}l} {1,} \hfill & {{\text{if}}\;{\text{task}}\;v\;{\text{is}}\;{\text{scheduled}}\;{\text{immediately}}\;{\text{after}}\;u} \hfill \\ {0,} \hfill & {{\text{otherwise}}} \hfill \\ \end{array} } \right.$$

The above formula indicates that if task *u* can be executed only after task *u* is executed (task *u* is called the predecessor task of task *v*). Then $J_{uv} = 1$, otherwise the value is 0. When a task has two or more predecessor tasks, the task can be completed only after all the predecessor tasks are executed^18^.

The virtual machine configuration algorithm proposed in this paper is to optimize the computational efficiency of the task, so it is necessary to establish a corresponding time model for the task time consumption. The execution time of a task is related to whether it is executed locally or migrated. Thus, this paper defines a binary quantity $I_{v} \in \left\{ {0,1} \right\}$ as the decision quantity for whether a task is migrated or not.

2 $$I_{v} = \left\{ {\begin{array}{*{20}l} {1,} \hfill & {{\text{if}}\;{\text{task}}\;v\;{\text{is}}\;{\text{executed}}\;{\text{at}}\;{\text{the}}\;{\text{local}}\;{\text{device}}} \hfill \\ {0,} \hfill & {{\text{if}}\;{\text{task}}\;v\;{\text{is}}\;{\text{executed}}\;{\text{at}}\;{\text{the}}\;{\text{nonlocal}}\;{\text{device}}} \hfill \\ \end{array} } \right.$$

The above equation indicates that if task *v* is executed locally, then $I_{v} = 1$; otherwise, $I_{v} = 0$. Tasks that must be performed locally can only be done locally.

1. When task *v* is executed locally, i.e., $I_{v} = 1$, its elapsed time is:
  3 $$T_{v}^{l} = a_{v} f_{l}^{ - 1}$$

In formula (3), $a_{v}$ represents the amount of computation of task *v* (unit: CPU cycles), which is proportional to the size of the computed task; $f_{l}^{{}}$ is the execution rate of the local CPU (unit: CPU cycles/s).

(2) When task* v* is migrated, i.e., $I_{v} = 0$, its execution time is:
  4 $$T_{v}^{c} = a_{v} f_{c}^{ - 1}$$

In the formula (4), $f_{c}^{{}}$ is the execution rate (unit: CPU cycles/s) of the CPU to which the task *v* is migrated.

(3) Data Transmission time
  5 $$T_{uv} = \left\{ {\begin{array}{*{20}l} {a_{uv} R_{s}^{ - 1} + T_{link} {,}} \hfill & {I_{u} = 1\& I_{v} = 0} \hfill \\ {a_{uv} R_{r}^{ - 1} + T_{link} {,}} \hfill & {I_{u} = 0\& I_{v} = 1} \hfill \\ \end{array} } \right.$$

In the above formula, $R_{s}$ and $R_{r}$ respectively represent the channel rate of data upload and the channel rate of data download (unit: bits/s). $T_{link}$ is the link delay, which represents the time spent by a single data packet sent by a physical machine to reach another physical machine through the transmission of switches at all levels, and is related to the number of switches passing through and the link status^19^. Among them, the transmission time of task migration is far greater than the execution time after migration.

(4) The task execution time in the case of considering task migration can be obtained:
  6 $$T(I) = \sum\limits_{v \in V} {\left[ {l_{v} T_{v}^{l} + (1 - I_{v} )T_{v}^{c} } \right]} + \sum\limits_{(u,v) \in A} {\mathop {\max }\limits_{u \in V} J_{uv} \left| {I_{u} - I_{v} } \right|T_{uv} }$$
  7 $$s.t.\quad I = [I_{1} ,I_{2} , \ldots ,I_{v} , \ldots ,I_{N + M} ]$$
  8 $$I_{v} \in \left\{ {0,1} \right\}$$

In formula (6), the first term on the right side of the equation represents the execution time of all tasks, which can be divided into two cases: local execution and migration to other areas for execution; the second term on the right side of the equation represents the time consumed for data transmission. Where $J_{uv}$ is a multiplicative factor indicating that the computation of task* v* will not start until the predecessor task *u* is completed^20^. In Eq. (7), *N* is the number of migration tasks; *M* is the number of tasks that must be executed locally; and *I* represents the execution position of each task.

### System addressing example

**Figure 2:**
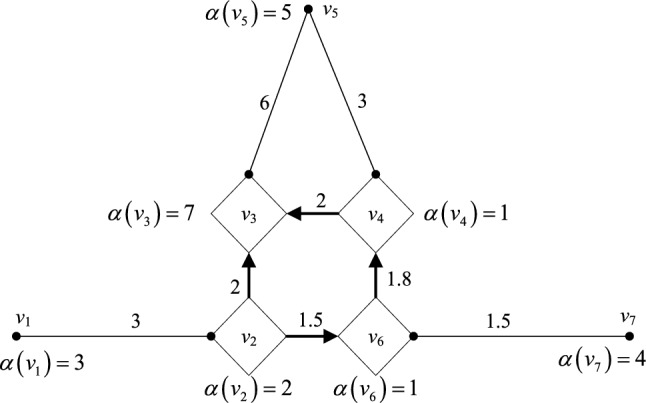
Network diagram of interconnected power grid.

The interconnected power grid in a certain area has 7 divisional power grids, and its network diagram is shown in Fig. 2. The vertex represents each partition; the vertex weight represents the number of power intelligent devices in each of the seven partition power grids; and the arc weight represents the bandwidth resource, namely, communication capability, between the seven power grid partitions.

1. The shortest path length $d_{ij} (i,j = 1,2, \ldots ,7)$ from each vertex $v_{i}$ to each other vertex $v_{j}$ in the above figure is obtained by using the Dijkstra algorithm, and the result is expressed as the following distance matrix *D*:
  9 $$D = \left[ {\begin{array}{*{20}c} {d_{11} } & {d_{12} } & \cdots & {d_{16} } & {d_{17} } \\ {d_{21} } & {} & {} & {} & {d_{27} } \\ \vdots & {} & \ddots & {} & \vdots \\ {d_{61} } & {} & {} & {} & {d_{67} } \\ {d_{71} } & {d_{72} } & \cdots & {d_{76} } & {d_{77} } \\ \end{array} } \right]$$
2. Obtain the weighted sum *A* of the shortest path lengths from each vertex to other vertices by using the weight $SVV(i) = D \times A \, (i = 1,2, \ldots ,7)$ of each vertex.
3. Judge the vertex of $\mathop {\max }\limits_{i} \left\{ {SVV(v_{i} )} \right\}$. Since the target partition is the point with the highest degree of electrical coupling with each partition, the result of $SVV(i)(i = 1,2, \ldots ,7)$ for each vertex is judged to select the vertex of $\mathop {\max }\limits_{i} \left\{ {SVV(v_{i} )} \right\}$ as the best placement position^21^. Select point $v_{1}$, that is, assign the calculation task of the coordination partition to partition 1 for calculation.

## Two-stage heuristic algorithm

**Figure 3:**
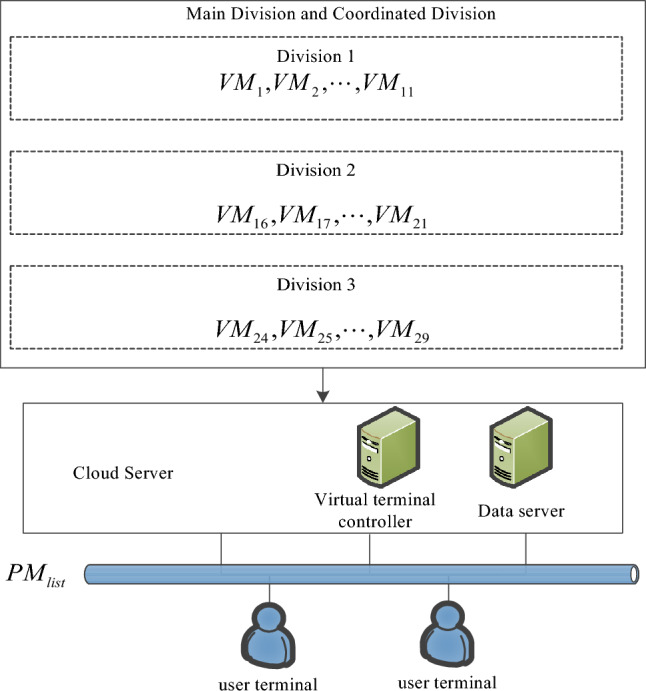
Virtual machine serial delivery.

A series of virtual machine sequences are obtained through the virtual machine sorting algorithm based on the critical path and the minimum cut. The next task is to study how to put these sequences into the physical machines with specific topology connections in the data center, as shown in Fig. 3.

When launching across physical machines or partitions, there are two situations that may cause virtual machines with close traffic relationships to be configured in different physical machines. One is cross-physical machine configuration, for example, when a physical machine configures virtual machine $VM_{16}$ in sequence *B*, there is no space to configure $VM_{17}$, and $VM_{17}$ can only be configured in an adjacent physical machine^22^. The other is cross-virtual machine sequence configuration. It is assumed that when configuring the virtual machine of sequence *A*, the remaining space of a physical machine is not enough to accommodate any virtual machine of sequence *A*. At this time, it is necessary to search for virtual machines that can be accommodated in other partitions. It is assumed that virtual machine $VM_{16}$ is found and $VM_{16}$ is forcibly configured in the physical machine^23^.

Since the more the number of nodes in the partition is, the more the traffic is transmitted, and the total amount of traffic in each partition is proportional to the number of nodes in the partition. The sequence of each partition is sorted as a whole according to the node number of each partition to obtain *A*, *B*, *C*. sequence virtual machine^24^.

## Experimental test analysis

### Experimental example

**Figure 4:**
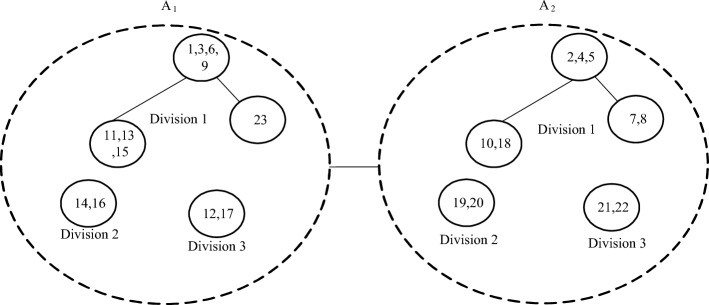
Regional power grid zoning.

The experiment in this paper is to partition the regional power grid A1 and A2 reasonably by using the decomposition and coordination algorithm, and then configure the virtual machine by using the two-stage heuristic algorithm to meet the needs of local regional task computing. In order to clearly compare the advantages and disadvantages of the algorithm proposed in this paper, the experiment in this paper does not consider the situation of task migration, that is, the local computing power is enough to complete the local computing task requirements^25^. The partition conditions and partition information inside the regional power grids A1 and A2 are described as follows:

The regional power grid A1 is an IEEE 30-node system, and the region is divided according to the tightness of electrical coupling, as shown in Fig. 4.

After the regional power grid A1 is divided into three sub-partitions, the specific data information inside each sub-partition is summarized as shown in Table 1.

**Table 1:** Information of each zone of regional power grid.

Zones	Number of generators	Number of transformers	Number of reactive compensation	Number of nodes
Zone one	2	0	1	12
Zone two	3	1	2	10
Zone three	1	1	1	8
Coordinator zone	0	0	0	12

### Analysis of experimental results

1. Comparative analysis of algorithm performance

The position of the coordination partition $A_{0}$ affects the operation speed of the whole system to some extent, and this paper calculates that the best placement position is in the partition $A_{3}$. In order to verify the effectiveness of the coordinated partition placed in the optimal partition in improving computational efficiency, comparative experiments were conducted using the methods in reference^6,7^ as comparative methods, with computational time and acceleration ratio as indicators. Among them, the acceleration ratio refers to how much performance improvement has been achieved compared to the runtime under the benchmark situation after using a certain optimization or parallel computing method. The higher the acceleration ratio, the more significant the optimization or parallel computing effect, and the more significant the performance improvement. Usually, the acceleration ratio should be greater than 1, indicating that the method or calculation result is obtained faster. The specific experimental results are shown in Table 2.

**Table 2:** Performance comparison of different configuration algorithms for coordination partition ($A_{0}$) placed in different partition power grids.

Number of regional power grid divisions	Configuration algorithm	A_0_ at A_1_		A_0_ at A_2_		A_0_ at A_3_	
		Computation time	Speed-up ratio	Computation time	Speed-up ratio	Computation time	Speed-up ratio
3	Reference^6^ methods	70.02	3.07	69.37	3.09	68.93	3.12
	Reference^7^ methods	53.88	4.01	53.16	4.08	52.76	4.17
	The method of this paper	48.19	4.42	47.69	4.47	47.01	4.50
4	Reference^6^ methods	62.73	3.39	62.23	3.45	61.29	3.51
	Reference^7^ methods	48.17	4.46	47.71	4.52	46.88	4.59
	The method of this paper	45.92	4.68	45.42	4.73	44.75	4.81

According to Table 2, when the number of regional power grid partitions remains unchanged, the same configuration algorithm is used to calculate tasks. Coordinate the different locations of zones in the regional power grid. The calculation time is also different. In the same regional power grid, when using different configuration algorithms to complete the same task calculation, the time required by this method is significantly less than the other two algorithms, and the acceleration ratio is much higher than the other methods. This is because the method in this article combines the characteristics of the system structure, and the system calculation coordination zone is located in the regional power grid with the highest degree of electrical coupling and strong computing power with other regions, which can optimize the system calculation efficiency. When the coordination partition is in the optimal partition, the bandwidth and computing power of the optimal partition can more effectively meet the needs of frequent information transmission and feedback of calculation results between partitions.

(2) Comparison between local computing and cloud computing.

**Figure 5:**
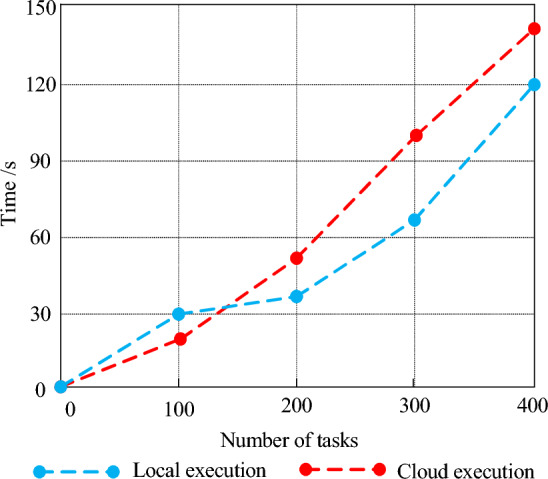
Computation time consumption of local computing and cloud computing under different number of tasks.

Compared with centralized cloud computing, the most obvious difference of edge computing is that edge computing is closer to the data source side, which uses the local edge site with certain storage, computing and communication capabilities to complete the calculation and processing of local data nearby. It avoids the transmission of data to the cloud after centralized collection, and then returns the calculation results to the local. It can save more task computing time^26^. In order to verify the effectiveness of this view, 100–400 tasks were set up. Local and remote computing were used to conduct experiments, and the task completion time was recorded and the results were plotted as shown in Fig. 5.

In Fig. 5, when the number of tasks is small, the difference between the task completion time of local computing and that of cloud computing is not significant. The task completion time of local computing is only about 10.11% less than that of cloud computing, because when the number of tasks is small, there is enough bandwidth for data transmission to ensure the efficiency of task transmission. The advantage of local computing is less obvious. However, with the increasing number of tasks, bandwidth pressure appears. The transmission time of tasks increases significantly. The task completion time of local computing is significantly less than that of cloud computing, which is about 28.56%. When the number of tasks exceeds 400, the local computing time increases significantly. With the increase of the number of tasks, the advantage of local computing is no longer obvious. The advantage of cloud computing begins to appear. This is because the local computing capacity is limited. When the computing capacity of local devices is exceeded, task migration will also cause a lot of transmission delay. The advantage of stronger cloud computing capacity leads to shorter task computing time.

**Figure 6:**
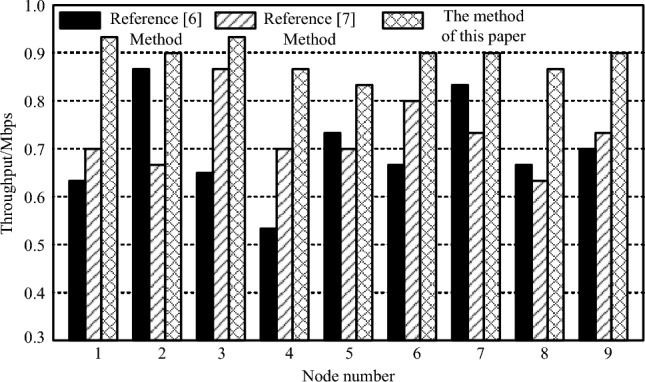
Throughput comparison results of different scheduling methods.

Throughput is another important indicator that reflects the performance of power system low delay resource scheduling model based on edge computing nodes. It refers to the amount of data successfully sent to other devices in the power system in unit time. The higher the throughput, the more reasonable the scheduling method. The throughput test results of different methods are shown in Fig. 6.

As shown in Fig. 6, the throughput of all three algorithms exceeds 0.5 Mbps, which can meet the basic scheduling needs of the power system. Among them, the throughput of the method in this article always remains around 0.9 Mbps, while the throughput performance of the other two methods is not stable enough. This indicates that the method proposed in this paper has superiority in meeting basic power system scheduling needs. It can stably provide high throughput, ensuring efficient computation when processing large amounts of data. In contrast, the other two methods may be influenced by some factors, leading to fluctuations or instability in throughput, which may affect the efficiency and reliability of power system scheduling.

## Conclusion

In this paper, according to the regional characteristics of interconnected power grids and the different degree of coupling between electricity, as well as the characteristics of edge computing platforms, a two-stage heuristic algorithm is proposed based on the traditional configuration algorithm, which is independent of the parallel algorithm of power system and can improve the computing efficiency of power system at the hardware level.

1. According to the urgency of the power system tasks, the tasks are divided into the tasks that must be executed locally and the tasks that can be migrated. Based on that, a time model is built to minimize the time consumption of task calculation.
2. Two of the most commonly used traditional virtual machine configuration algorithms, descending best fit algorithm and hierarchical clustering algorithm, are analyzed. Combined with the characteristics of power system, a two-phase heuristic algorithm for power system parallel distributed computing is proposed.
3. Without considering the task migration, the decomposition and coordination algorithm isused to partition the regional power grid. The effect of the proposed algorithm is compared with the traditional descending best fit algorithm and hierarchical clustering algorithm in terms of computing time and energy consumption. In addition to considering the influence of regionality and partition control characteristics on electrical operation, the number of partitions and the uniformity of the number of nodes between partitions will also affect electrical operation. If the number of partitions is too small, the speedup of electrical parallel operation will be small; if the number of partition nodes is too large, the iteration times of electrical parallel operation will be increased, and the results may not converge. Therefore, how to determine the number of partitions and the calculation scale is a key issue to be studied.

## Data Availability

The datasets used and/or analysed during the current study available from the corresponding author on reasonable request.
